# Effect of Diets Containing Phytoestrogen on Livestock Production: Nutrient Utilization, Carcass Traits, Lactational Performance, and Reproductive Function—A Review

**DOI:** 10.3390/molecules31101724

**Published:** 2026-05-19

**Authors:** Sina Salimolnafs, Maghsoud Besharati, Deniz Azhir, Lucrezia Forte, Pasquale De Palo, Eric N. Ponnampalam, Abdelfattah Z. M. Salem, Aristide Maggiolino

**Affiliations:** 1 Department of Animal Science, Ahar Faculty of Agriculture and Natural Resources, University of Tabriz, Tabriz 51666, Iran; 2 Department of Food Science and Technology, Faculty of Agriculture, University of Tabriz, Tabriz 51666, Iran; 3 Department of Veterinary Medicine, University of Bari Aldo Moro, 70010 Bari, Italy; lucrezia.forte@uniba.it (L.F.);; 4 School of Agriculture, Food and Ecosystems Sciences, The University of Melbourne, Parkville, VIC 3010, Australia; eponnampalam@unimelb.edu.au; 5 Formerly Agriculture Victoria Research, Department of Jobs, Precincts and Regions, Bundoora, VIC 3083, Australia; 6 Department of Soil, Plant and Food Sciences, University of Bari Aldo Moro, 70126 Bari, Italy

**Keywords:** phytoestrogen, livestock, cattle, performance, meat production, milk production

## Abstract

Phytoestrogens are plant-derived phenolic compounds that structurally resemble endogenous estrogens and can exert both estrogenic and anti-estrogenic effects in animals. In ruminant nutrition, the main classes of phytoestrogens (isoflavones, lignans, stilbenes, coumestans and selected flavonoids) are supplied predominantly by legume forages and soybean-based feeds, in which concentrations can reach several mg/g of dry matter. After ingestion, these compounds are extensively metabolized by the rumen microbiota to derivatives with altered biological potency, such as equol and p-ethyl-phenol, which influence endocrine, immune and metabolic pathways. Experimental and field studies in cattle, sheep and goats indicate that dietary phytoestrogens may improve nitrogen utilization, immune competence, growth performance, antioxidant status and milk yield. However, they can also impair fertility, modify hormone profiles and compromise embryo survival in a compound-, dose-, and species-dependent manner. In this review, we summarize current knowledge on the botanical and nutritional sources, ruminal metabolism and transfer of phytoestrogens in ruminants, and critically examine their effects on blood metabolites, immune responses, growth and carcass traits and lactational performance and reproductive function. A structured literature search based on PRISMA principles was used to identify and appraise experimental and observational studies in both grazing and intensive production systems up to 2025. Remaining knowledge gaps and practical implications for the safe use of phytoestrogen-rich feeds in livestock production are highlighted.

## 1. Introduction

Global demand for animal-source foods continues to increase, placing pressure on ruminant production systems to improve efficiency while maintaining animal health and product quality. In parallel, concerns about antimicrobial resistance and food safety have led many countries to restrict or ban the prophylactic use of antibiotic growth promoters, stimulating interest in phytogenic feed additives as natural alternatives to support productivity and health. Within this broader group of plant secondary metabolites, phytoestrogens represent a structurally and functionally distinct subset with a strong affinity for estrogen receptors and a unique potential to influence both performance and reproductive function in livestock. The term “phytoestrogen” refers to plant-derived phenolic compounds that can bind to estrogen receptors and exert estrogenic or anti-estrogenic activities. They are commonly grouped into isoflavones, lignans, stilbenes, coumestans and selected flavonoids [1,2]. In botanical systems, phytoestrogens act primarily as phytoalexins, accumulating in response to environmental stressors and microbial attack and contributing to plant defence through antifungal, antibacterial and antioxidant properties [3]. In ruminant nutrition, the main sources of phytoestrogens are legume forages such as red and white clover (*Trifolium repens* L.) and lucerne, soybean (*Glycine max* (L.) Merr.) and soybean by-products, and flaxseed (*Linum usitatissimum* L.) and other oilseeds, in which concentrations of isoflavones and lignans can reach several mg/g of dry matter [4,5,6]. After ingestion, phytoestrogens and their conjugates are extensively transformed by the rumen microbiota into metabolites with altered biological potency, including equol and p-ethyl-phenol from isoflavones and enterolactone from lignans. These metabolites can modulate endocrine, immune and metabolic pathways and are transferred to milk and other animal products. Beyond their endocrine actions, phytoestrogens exhibit antimicrobial, antioxidant, anti-inflammatory and other bioactivities in animal tissues, although their effects are generally weaker than those of 17β-estradiol [2,7,8,9]. Field observations and experimental studies in ruminants have shown that moderate exposure to phytoestrogen-rich feeds may improve antioxidant status, nitrogen utilization, growth performance and milk yield, whereas prolonged or high exposure, particularly to highly estrogenic clovers, has been associated with reduced fertility, increased embryonic loss and structural lesions in estrogen-sensitive organs, especially in sheep [10,11,12,13]. Phytoestrogen concentrations in plants are highly variable and depend on species and cultivar, stage of growth, environmental conditions, fertilization, disease and conservation method. Management of grassland and forage systems therefore plays a key role in determining the phytoestrogen intake of grazing ruminants and the levels of phytoestrogen metabolites in milk and other products [14,15]. This complexity highlights the need to consider phytoestrogens as bioactive feed components whose effects depend on dose, chemical form, plant source, length of exposure and species sensitivity, rather than as uniformly beneficial or harmful compounds. In this context, the present review focuses on the effects of feeding phytoestrogen-containing forages, concentrates and purified compounds to livestock, with emphasis on ruminant species. Building on previous work on phytoestrogen chemistry, metabolism and toxicology, we synthesize experimental and field evidence on their impacts on growth and performance, milk production, reproductive function, immune responses and blood metabolism. Particular attention is paid to dose–response relationships, differences among phytoestrogen classes and plant sources, and knowledge gaps relevant for practical feeding strategies and risk assessment.

## 2. Review Design

This work was conceived as a structured narrative review with elements of a scoping review, aimed at collating and critically appraising experimental and observational evidence on the effects of dietary phytoestrogens in livestock, with emphasis on ruminant species. The search, screening and reporting process followed the general principles of the PRISMA 2020 statement for systematic reviews and of the PRISMA extension for scoping reviews (PRISMA-ScR) [16,17]. A comprehensive literature search was performed in the electronic databases PubMed, Scopus, Web of Science Core Collection and CAB Abstracts. Google Scholar was consulted to identify additional or grey literature and to cross-check for potentially missed articles. No a priori limits on publication year were applied; all records available in each database up to October 2025 were considered. The search strategy combined controlled vocabulary (where available) and free-text terms related to phytoestrogens and livestock species. Core terms encompassed compound-related keywords (“phytoestrogen”, “isoflavone”, “formononetin”, “daidzein”, “genistein”, “biochanin A”, “coumestrol”, “lignan”, “resveratrol”, “stilbene”) combined with species- and system-related terms (“ruminant”, “cattle”, “cow”, “dairy”, “beef”, “bovine”, “sheep”, “ewe”, “ram”, “goat”, “caprine”, “buffalo”, “livestock”, “farm animal”) and feeding-related terms (“feed”, “diet”, “pasture”, “forage”, “hay”, “silage”, “concentrate”). Search strings were adapted to the syntax of each database and refined iteratively to balance sensitivity and specificity. Reference lists of key primary studies and recent reviews were screened manually to identify additional relevant publications. Studies were eligible when they met the following criteria: (i) population: livestock species, mainly ruminants (cattle, sheep, goats, buffalo), with other farm animals (e.g., pigs, poultry) included only when directly relevant to the objectives of this review; (ii) intervention/exposure: intake of phytoestrogen-containing plant materials (e.g., clovers, lucerne, soybean and other legumes), phytoestrogen-rich extracts or purified compounds supplied via the diet or drinking water; (iii) comparator: control diets or management conditions differing in the presence or level of phytoestrogens, including comparisons among botanical compositions, conservation methods or legume inclusion levels; (iv) outcomes: at least one relevant response related to growth and performance, lactational performance and milk composition (including transfer of phytoestrogens and metabolites into milk), reproductive function and fertility, immune or antioxidant responses, health indicators, or blood metabolites and endocrine variables associated with energy and protein metabolism. Exclusion criteria comprised studies conducted exclusively in vitro, ex vivo or in laboratory animals or humans (except when used only as mechanistic background), studies using non-nutritional routes of phytoestrogen administration (e.g., implants, injections) without a dietary component, non-peer-reviewed material (e.g., theses, non-refereed reports), conference abstracts without full text, editorials and commentaries, and papers not available in English. Narrative and systematic reviews and meta-analyses were not used as primary sources of evidence, but their reference lists were screened for additional eligible studies. All records retrieved from database searches were exported to a reference manager and duplicates were removed. Study selection was performed in two stages: initial screening of titles and abstracts to exclude clearly irrelevant records, followed by full-text assessment against the eligibility criteria. Disagreements were resolved by discussion among the authors. For each included study, data were extracted using a structured form covering bibliographic information, country, species and physiological category, production system, study design and sample size, diet composition and phytoestrogen source(s), analytical methods and dietary concentrations of major phytoestrogen classes and compounds, duration of exposure and main outcomes in the predefined response categories. Owing to substantial heterogeneity in species, diets, phytoestrogen profiles, doses, exposure length and outcome measurements, no quantitative meta-analysis was attempted. Instead, a qualitative synthesis was undertaken, grouping studies by phytoestrogen source, response category and livestock species to identify consistent patterns, dose–response relationships and remaining knowledge gaps.

## 3. Classification of Phytoestrogens

Phytoestrogens are non-steroidal phenolic compounds that can interact with estrogen receptors and elicit estrogen-like or anti-estrogenic responses. Different classification systems have been proposed, but for nutritional and physiological purposes in livestock it is most useful to distinguish five main structural groups: isoflavones, other flavonoids, stilbenes, coumestans and lignans [1,11,18,19]. These classes comprise more than one hundred individual molecules, which differ in the number and position of hydroxyl, methoxy and other substituents on a common C_6_–C_3_–C_6_ or C_6_–C_2_–C_6_ backbone, and in the extent of glycosylation or prenylation. Representative compounds within each class are summarized in Table 1.

Among phytoestrogens relevant to ruminant diets, isoflavones and lignans are generally the quantitatively most important, because they occur at relatively high concentrations in legume forages, soybean products and oilseeds, whereas coumestrol and related coumestans can become critical under specific agronomic or plant health conditions (e.g., diseased lucerne and clovers). Flavonoids overall tend to display higher estrogenic potency than lignans in in vitro assays [21], but effective activity in vivo depends strongly on rumen biotransformation and on the formation of metabolites such as equol and enterolactone, which are discussed in the following sections.

### 3.1. Isoflavones

Isoflavones are diphenolic compounds structurally related to 17β-estradiol, characterized by a 3-phenylchroman backbone with the B ring at position 3 of the heterocycle. The main dietary isoflavones relevant to livestock are daidzein, genistein, glycitein, formononetin and biochanin A, together with their glycosylated and acylated conjugates [4,22]. In plants, isoflavones occur predominantly as β-glycosides (e.g., daidzin, genistin, ononin, sissotrin) or as acetyl- and malonylglycosides, whereas the corresponding aglycones are present in lower proportions [11,23]. The main aglycones and their glycosides, including the pattern of hydroxylation and methoxylation on the A and B rings, are summarized in Table 2.

In ruminant diets, isoflavones are supplied mainly by legume forages and soybean products. Soybeans and soybean meal contain daidzein, genistein and their conjugates at concentrations typically in the range of 1.2–4.2 mg/g dry matter (DM), with considerable variation among cultivars and growing conditions [4]. Red clover (*Trifolium pratense*) is particularly rich in isoflavones, predominantly formononetin and biochanin A, and can reach total isoflavone concentrations of 10–25 mg/g DM, whereas white clover usually contains only 0.5–0.6 mg/g DM [24,25]. Lucerne/alfalfa (*Medicago sativa*) generally contains 0.05–0.3 mg isoflavones/g DM [26]. More comprehensive values for different plant species and their isoflavone profiles are reported in Table 3, while representative concentrations of major isoflavones (and selected lignans) in common food and feed sources are presented in Table 4 [1,27,28].

**Figure 1:**
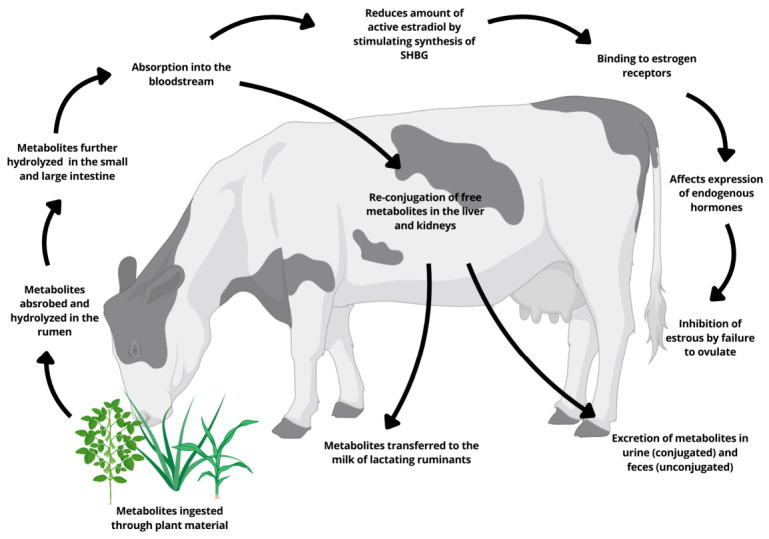
The metabolic processes involving isoflavones and coumestans in ruminants under grazing conditions [18]. SHBG = sex hormone-binding globulin.

**Figure 2:**
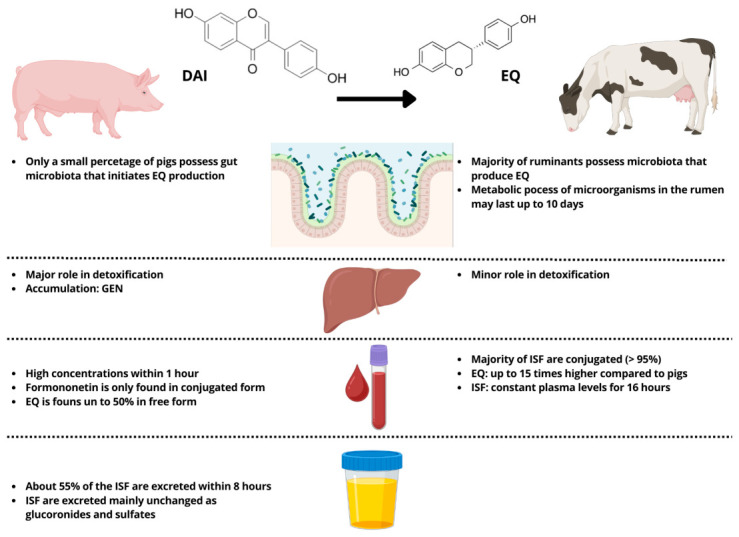
Comparative metabolic profiles of isoflavones in pigs and ruminants. The left side shows pigs and the right side shows ruminants. ISF = isoflavones; DAI = daidzein; EQ = equol; GEN = genistein. Adapted from [34]. Created with BioRender.com. Maggiolino A. (2026).

Following ingestion, isoflavone glycosides are rapidly hydrolysed in the rumen to release the aglycones daidzein, genistein, formononetin and biochanin A, which are then further transformed by ruminal microorganisms into a range of metabolites. The most important from an endocrine standpoint is S-equol, derived from daidzein and formononetin via dihydrodaidzein, together with O-desmethylangolensin and ring-cleavage products such as p-ethyl-phenol that lack estrogenic activity [11,29,30,31]. Ruminants are generally efficient equol producers, and equol has a higher affinity for estrogen receptors and a longer elimination half-life than its precursors, making it a key determinant of systemic estrogenic exposure. The details of ruminal biotransformation, absorption and transfer of isoflavones and their metabolites are discussed in the dedicated section on metabolism. After hydrolysis of the glycosides, the released aglycones are extensively biotransformed by ruminal microorganisms. Isoflavones present in soybean (genistein, daidzein and glycitein) and in red clover (formononetin, daidzein, biochanin A and genistein) are mainly converted to S-equol and, to a lesser extent, to O-desmethylangolensin and ring-cleavage products such as p-ethyl-phenol, which lack estrogenic activity [11,29,30,31]. Demethylation of biochanin A yields genistein, whereas formononetin is predominantly demethylated to daidzein before further hydrogenation and ring-cleavage products. In vitro incubations in bovine rumen fluid indicate half-lives of approximately a few hours for formononetin and biochanin A, confirming that the rumen is a major site of isoflavone degradation [30]. Only a small fraction of the parent compounds escapes the rumen: in dairy cows, apparent absorption of biochanin A and genistein in the omasum is low (0–9%), whereas formononetin and daidzein show higher apparent absorption (7–16%), and about 12% of the daily intake of daidzein and formononetin can be detected in the abomasum [32,33]. The post-ruminal fate of isoflavones and coumestans in grazing ruminants is illustrated in Figure 1, whereas Figure 2 compares the main metabolic pathways in ruminants and pigs, underlining both the central role of rumen fermentation and the species differences in equol production and systemic exposure.

### 3.2. Flavonoids

**Figure 3:**
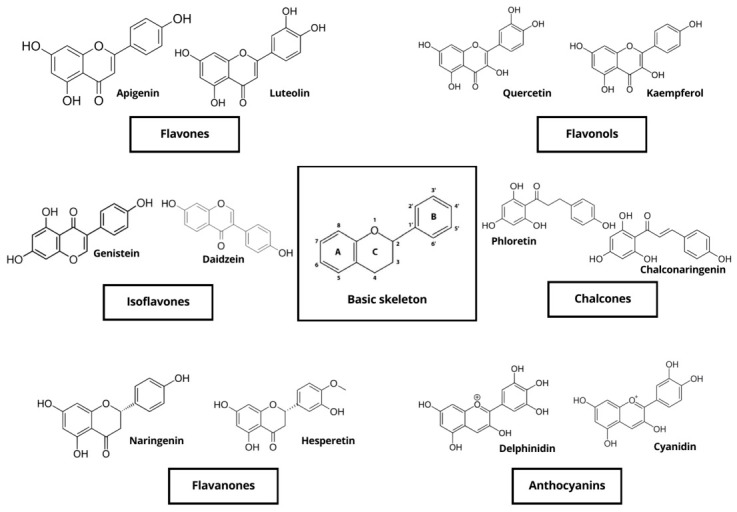
Basic skeleton structure of flavonoids and their classes.

Flavonoids are a large group of low-molecular-weight polyphenolic compounds derived from the phenylpropanoid pathway and characterized by a C_6_–C_3_–C_6_ backbone consisting of two aromatic rings linked by a heterocyclic pyran ring; their basic chemical skeleton and main subclasses are shown in Figure 3.

They are usually divided into flavones (e.g., apigenin, luteolin), flavonols (e.g., quercetin, kaempferol), flavanones (e.g., naringenin, hesperetin), flavan-3-ols and proanthocyanidins (e.g., catechin, epicatechin), anthocyanidins and prenylated flavonoids (e.g., xanthohumol, 8-prenylnaringenin) [35,36,37]. A subset of these compounds is recognized as phytoestrogenic because it can bind to estrogen receptors and modulate estrogen-dependent signalling pathways [37,38,39]. In plants, flavonoids contribute to UV protection, pigmentation, signalling and defence against pathogens and herbivores, and they occur mainly as O- or C-glycosides with sugars attached at positions 3 or 7, which strongly influences solubility, stability and bioavailability [40,41]. As with isoflavones, dietary flavonoid glycosides ingested by ruminants are largely hydrolysed by rumen microorganisms, releasing the corresponding aglycones that can then be further metabolized, absorbed or degraded [42]. Particular attention has been given to prenylated flavonoids, which combine a flavanone or chalcone core with one or more prenyl groups. 8-Prenylnaringenin (8-PN), a constituent of hops (*Humulus lupulus* L.), is among the most potent phytoestrogens identified to date, showing high binding affinity and strong agonistic activity at both ERα and ERβ in vitro and in vivo [41,43,44,45]. Although concentrations of 8-PN in standard rations are usually low, exposure may occur when hop by-products or brewery residues are used as feed ingredients, which could be relevant for reproductive health in high-producing females. In practical ruminant nutrition, non-isoflavonoid flavonoids are supplied mainly through fresh and conserved forages, agro-industrial by-products and phytogenic feed additives. Citrus pulp, citrus peel and other citrus co-products provide flavanones such as naringin and hesperidin, whereas grape pomace (*Vitis vinifera* L.), grape seeds and winery residues are rich in flavan-3-ols, proanthocyanidins and other flavonoids [46,47,48]. Recent reviews indicate that these flavonoid-rich by-products can be incorporated into ruminant diets without impairing feed intake and may improve nutrient utilization, oxidative status and product quality, while also supporting circular economy strategies [46,48,49]. Several experimental studies and meta-analyses have evaluated the effects of dietary flavonoids on rumen fermentation, performance and health in cattle and small ruminants. Supplementation with flavonoid-rich extracts or by-products has been associated with improved average daily gain and feed efficiency, enhanced digestibility, and reductions in markers of oxidative stress and systemic inflammation [50,51,52]. Flavonoids can modulate ruminal volatile fatty acid profiles, reduce methane formation and mitigate the decline in rumen pH in high-concentrate diets, partly through selective antimicrobial effects on rumen bacteria and protozoa [42,50]. These actions support the concept of flavonoids as multifunctional feed additives with potential to improve productivity, health and environmental sustainability in ruminant systems. Regarding endocrine effects, only a subset of flavonoids is likely to reach systemic concentrations in livestock compatible with marked estrogenic or anti-estrogenic activity. In vitro assays demonstrate that apigenin, naringenin, quercetin, kaempferol and resveratrol can activate estrogen-responsive reporter systems and modulate progesterone receptor expression, confirming their ability to interact with estrogen signalling [35,53,54]. However, most in vivo studies using flavonoid-rich feeds in ruminants report beneficial effects on performance, immune function and oxidative status without clear evidence of impaired fertility, suggesting that at typical inclusion levels their endocrine activity is limited compared with that of isoflavones and coumestans [18,50]. Nevertheless, potential additive or synergistic interactions between different flavonoid subclasses and other phytoestrogens, particularly in high-legume pasture systems, cannot be excluded and warrant further investigation. Overall, flavonoids constitute a structurally and functionally heterogeneous group of phytoestrogens whose antioxidant, antimicrobial and rumen-modulating properties are generally beneficial, but which may also contribute to the overall estrogenic load of the diet and should therefore be evaluated with both productive and reproductive endpoints in mind.

### 3.3. Stilbenes

**Figure 4:**
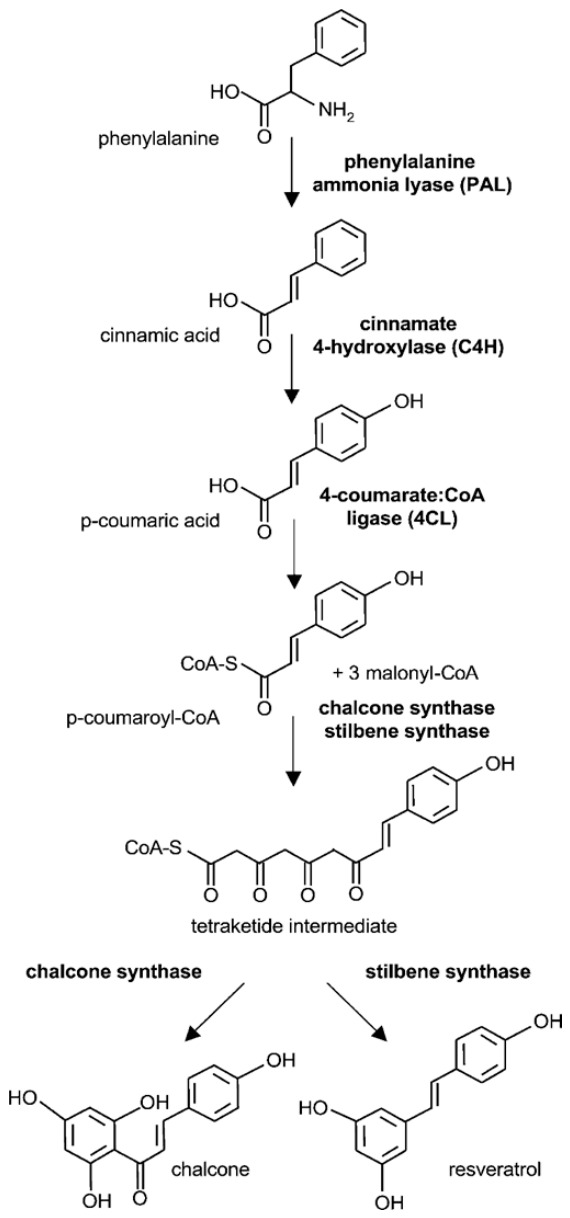
Early steps of stilbene biosynthesis.

Stilbenes are a class of polyphenolic compounds derived from the phenylpropanoid pathway and characterized by a 1,2-diphenylethylene (C_6_–C_2_–C_6_) backbone. The basic stilbene scaffold can be variously hydroxylated, methoxylated, prenylated or glycosylated, giving rise to a wide range of derivatives, of which resveratrol, pterostilbene and piceid are the most extensively studied [28,55,56]. The early steps of stilbene biosynthesis from phenylalanine and cinnamic acid are summarized in Figure 4, and selected dietary stilbenes together with their structural variants and botanical occurrence are reported in Table 5.

In plants, stilbenes act mainly as phytoalexins, inducible defence compounds synthesized in response to biotic and abiotic stress. Important dietary sources include grapes and grape products, berries, peanuts and certain tree species; in livestock nutrition, stilbenes enter the diet primarily via grape pomace and winery by-products, specialized phytogenic additives or experimental supplementation with purified resveratrol [58,59]. Because of their structural similarity to endogenous estrogens, several stilbenes are recognized as phytoestrogens. Trans-resveratrol binds to ERα and ERβ and modulates genomic and non-genomic signalling, showing mixed agonist/antagonist activity in a cell- and context-dependent manner [60,61,62,63]. In vitro assays confirm measurable estrogenic activity of resveratrol and related stilbenes in estrogen-responsive cell systems, albeit weaker than 17β-estradiol and often accompanied by antiproliferative or cytotoxic effects at higher concentrations [64,65]. Pterostilbene, a dimethylated analogue of resveratrol found in blueberries and some other plants, also signals via ER-dependent pathways and may require ERβ for some cellular actions, reinforcing the classification of stilbenes as phytoestrogen [66,67,68,69]. Beyond their endocrine properties, stilbenes exert antioxidant, anti-inflammatory, metabolic and epigenetic effects, modulating pathways such as NF-κB and MAPK and influencing sirtuin activity and redox balance [55,56,59]. In ruminant nutrition, most work has focused on resveratrol as a model stilbene. In vitro studies show that resveratrol can modify rumen fermentation patterns, altering pH, volatile fatty acid profiles and microbial communities, with implications for fibre degradation, methane production and nitrogen utilization [70,71,72,73]. In vivo trials and recent reviews in dairy cows and small ruminants indicate that low-to-moderate dietary doses of resveratrol can improve indicators of oxidative status, reduce markers of systemic inflammation and, in some cases, support performance and metabolic homeostasis, particularly around the transition period [58,74]. From an endocrine perspective, the contribution of stilbenes to the overall estrogenic load of typical ruminant diets is probably small compared with that of isoflavones and coumestans, because their concentrations in conventional feeds are low and most estrogenicity data derive from cell or non-ruminant models. Nevertheless, the clear ability of resveratrol and pterostilbene to interact with estrogen receptors suggests that their potential endocrine effects should be considered when high doses of purified stilbenes are used as feed additives, especially in breeding females or during critical reproductive windows.

### 3.4. Lignans

**Figure 5:**
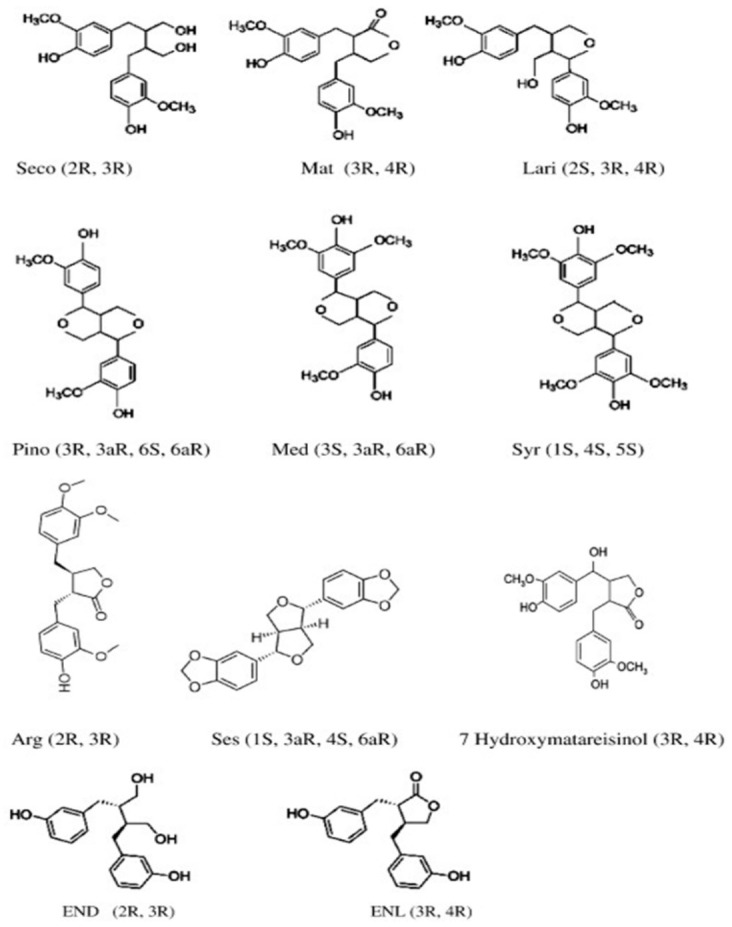
The following compounds are classified as plant lignans: Mat: matairesinol, Seco: secoisolariciresinol, Med: medioresinol, Lari: lariciresinol, Pino: pinoresinol, Arg: artigenin, Syr: syringaresinol, and Ses: sesamin. Additionally, the mammalian lignans include END: enterodiol and ENL: enterolactone.

Lignans are phenolic dimers formed by oxidative coupling of two coniferyl alcohol-derived C_6_–C_3_ units of the phenylpropanoid pathway. They share a 2,3-dibenzylbutane skeleton with variable patterns of oxidation, methoxylation and glycosylation. The most abundant plant lignans in foods and feeds include secoisolariciresinol (SECO), secoisolariciresinol diglucoside (SDG), matairesinol (MAT), pinoresinol (PIN), lariciresinol (LAR), medioresinol (MED), syringaresinol (SYR), arctigenin and sesamin [75,76,77]. After ingestion, plant lignans are converted by intestinal or ruminal microbiota into the so-called enterolignans or mammalian lignans, principally enterodiol (ED) and enterolactone (EL), which are responsible for most systemic effects in mammals [75,78]. Representative plant and mammalian lignans are illustrated in Figure 5.

Lignans are widely distributed in seeds, cereals, legumes, vegetables, fruits and beverages such as coffee and tea, and represent the predominant phytoestrogens in Western human diets [75,76]. For livestock, however, the richest practical source is flaxseed (linseed), in which the major lignan SDG is concentrated in the fibrous outer layers of the seed. Flaxseed can contain several grams of SDG per kilogram of dry matter, making it by far the main lignan source used in ruminant feeding [75,78]. Whole flaxseed, flaxseed meal, hulls and cakes are therefore key ingredients when considering lignan intake in dairy and beef cattle, sheep and goats, whereas grasses and most conventional forages typically contain much lower lignan concentrations [32,78]. The lignan content and composition of key plant species relevant to ruminant diets are summarized in Table 6.

In ruminants, the rumen is the main site of lignan biotransformation. SDG from flaxseed is first deglycosylated to SECO, which is then reduced and dehydrogenated by ruminal microorganisms to form ED and subsequently EL [32,79,80]. In vivo studies with rumen-cannulated dairy cows and goats show that rumen microbiota efficiently convert SDG to enterolignans and that this process can affect volatile fatty acid profiles and microbial community composition [79,80]. Most EL appears to be formed in the rumen, absorbed through the rumen wall and then conjugated (mainly as glucuronides and sulfates) before being distributed via blood to urine and milk [78,80]. A distinctive feature of lignans compared with other phytoestrogens is this efficient transfer of EL to milk. Several trials in dairy cows and sheep have demonstrated that feeding flaxseed products (whole seed, hulls, meal, cakes) markedly increases EL concentrations in rumen fluid, plasma, urine and milk, with EL being the main mammalian lignan detected in milk [80,81,82,83]. Analytical advances, particularly LC-MS/MS, now allow simultaneous quantification of free and conjugated plant lignans and enterolignans in biological matrices, improving the assessment of lignan exposure in both animals and consumers of milk and dairy products [81]. From a functional standpoint, lignans and enterolignans exhibit antioxidant, anti-inflammatory and anticarcinogenic properties and have been associated with reduced risk of cardiovascular disease and hormone-dependent cancers in humans [75,76,77,78]. In ruminants, increased EL concentrations in blood and milk of cows fed flaxseed products have been linked to improved antioxidant capacity and some aspects of immune status, although data are still limited and sometimes inconsistent [78,84]. From an endocrine perspective, lignans are generally considered weaker phytoestrogens than isoflavones and coumestans. Enterolactone and enterodiol can bind to estrogen receptors and display selective estrogen receptor modulator (SERM)-like properties, but their affinity for ERα and ERβ is typically much lower than that of coumestrol or genistein [18,78]. At dietary inclusion levels commonly used to manipulate milk fatty acid profile or nutraceutical properties (e.g., 5–15% flaxseed products in the total ration), there is currently no strong evidence of adverse reproductive effects in dairy cows; most studies have focused instead on production responses and milk quality [83,85,86]. Nonetheless, reviews on phytoestrogens in livestock emphasize that high lignan intake, especially during sensitive windows such as gestation and early life, might theoretically influence developmental programming and should therefore be monitored [18,78]. Overall, lignans represent a structurally distinct, quantitatively important but comparatively weakly estrogenic class of phytoestrogens in ruminant diets. Their main relevance at present is linked to the use of flaxseed and related co-products to enrich milk in n-3 fatty acids and EL, with potential benefits for animal oxidative status and human health, while their endocrine activity and efficient transfer to milk mean they should be considered alongside isoflavones and coumestans when evaluating total phytoestrogen exposure in high-producing and breeding animals.

### 3.5. Coumestans

Coumestans are a small group of phenolic compounds structurally related to coumarins and isoflavones, characterized by a fused benzofurochromen-6-one ring system. The most relevant dietary coumestans are coumestrol and its methoxylated derivatives (e.g., 4′-methoxycoumestrol), which exhibit strong estrogenic activity and are therefore classified as potent phytoestrogens [87,88]. Coumestrol was first isolated as a new estrogen from white clover and strawberry clovers (*Trifolium fragiferum* L.) and alfalfa (*Medicago sativa* L.) in the 1950s, and its occurrence in forage crops was rapidly linked to impaired reproductive performance in grazing livestock [88]. In practical feeding conditions, coumestans occur mainly in legume forages, particularly lucerne/alfalfa, subterranean clover (*Trifolium subterraneum*), white clover and strawberry clover, but they have also been detected in soybean, other forage legumes and various vegetables [87,89]. Their concentration is highly variable and influenced by species, cultivar, stage of growth and, especially, environmental stress. Plant disease, fungal infection, insect damage and other stressors can markedly increase coumestrol accumulation, so diseased lucerne or clover stands may show much higher estrogenic activity than healthy plants [89,90]. In lucerne, coumestrol contents exceeding ≈40 mg/kg DM have been associated with reduced fertility in sheep and cattle, whereas values above 100 mg/kg DM are considered high-risk for clover disease [89,90]. From a mechanistic standpoint, coumestrol is among the most potent naturally occurring phytoestrogens. It binds to both ERα and ERβ with high affinity, in some assays approaching that of 17β-estradiol, and generally shows stronger estrogen receptor-binding activity than most isoflavones [91]. Comparative in vitro data indicate that the relative estrogenic activity of coumestrol, expressed as a percentage of 17β-estradiol, is higher than that of genistein, equol and daidzein, underlining its high intrinsic potency [1]. 

In vitro and in vivo studies confirm that coumestrol can elicit classical uterotrophic responses and modulate ER-dependent gene expression, acting predominantly as an ER agonist at low doses but also showing tissue-specific and dose-dependent antagonist or modulatory effects [91,92]. In ruminants, coumestans are ingested mainly as glycosides within the plant matrix. After ingestion, these glycosides are hydrolysed by rumen microbes to release the aglycone coumestrol, which appears to undergo less extensive degradation than many isoflavones and may therefore reach comparatively high systemic concentrations [32,93]. Available data suggest that coumestrol itself, rather than its metabolites, is the principal estrogenic entity in ruminants, in contrast to isoflavones where equol and other microbial metabolites often dominate biological activity [32,89]. The degree of ruminal conversion and absorption of coumestans can vary with the mode of ingestion (grazed versus cut-and-carried lucerne), the maturity and health status of the forage, and the adaptation of the rumen microbiota [90,93]. Historically, coumestans have been most intensively studied because of their role in clover disease and related reproductive disorders in sheep. Classical field observations in Western Australia and subsequent experimental studies showed that prolonged grazing of highly estrogenic subterranean clover pastures caused severe and often irreversible infertility in ewes, characterized by increased embryonic loss, reduced conception rates, cervical and uterine abnormalities, and dystocia [94]. Together with isoflavones, coumestans therefore represent the main contributors to diet-related estrogenic disorders in grazing ruminants, and monitoring their concentrations in high-legume pastures remains a key component of reproductive risk management.

## 4. The Effect of Phytoestrogens on Ruminant Nutrition

Dietary phytoestrogens can influence several aspects of ruminant nutrition, including feed intake, nutrient digestibility, rumen fermentation, nitrogen utilization, growth performance and lactational responses. Their net impact depends on botanical source, chemical class (isoflavones, coumestans, lignans, other flavonoids), dose, duration of exposure and production system (grazing vs. confined), and may range from neutral or beneficial to clearly detrimental when intake is excessive or reproductive endpoints are considered [18,34].

### 4.1. Effects on Feed Intake and Nutrient Utilization

Legume forages rich in phytoestrogens, particularly red clover and lucerne, are often associated with higher voluntary intake and improved nutritive value compared with grass-only forages, mainly because of their higher crude protein content, lower neutral detergent fibre and greater ruminal degradability of organic matter [25,34,95]. Dairy cows fed clover-grass silages containing red clover typically show increased dry matter intake and milk yield relative to animals fed grass only or green silages, although the specific contribution of phytoestrogens versus other legume attributes is difficult to disentangle [25,32]. Recent work has highlighted that red clover isoflavones, especially biochanin A and formononetin, may directly influence rumen nitrogen metabolism and thereby improve protein utilization efficiency. In vitro and in vivo studies show that red clover isoflavones can inhibit urease activity, decrease the abundance of urealytic bacteria, reduce ruminal ammonia-N concentrations and enhance microbial protein synthesis, ultimately increasing nitrogen utilization efficiency in dairy cows [96,97]. Similarly, supplementation of biochanin A in dairy cow diets increased microbial nitrogen flow and improved feed efficiency without negatively affecting total tract digestibility [34,96]. Flavonoid-rich plant extracts and agro-industrial by-products (e.g., citrus and grape co-products) have also been shown to modulate rumen fermentation and nutrient utilization. Meta-analyses and controlled trials indicate that flavonoid supplementation can increase total volatile fatty acid production, shift the acetate:propionate ratio, and sometimes reduce methane output, while maintaining or improving fiber digestibility [50,51,52]. These effects are partly attributed to selective antimicrobial activity on rumen bacteria and protozoa and to changes in the relative abundance of fibrolytic and amylolytic taxa [42,50]. Overall, the available evidence indicates that the effects of phytoestrogens on feed intake and nutrient utilization are closely linked to the nutritional quality of the plant source and to ruminal microbial metabolism. Moderate inclusion of phytoestrogen-rich legumes or flavonoid-rich by-products may improve rumen fermentation, nitrogen utilization and feed efficiency, whereas excessive or poorly controlled exposure should be interpreted cautiously, particularly when reproductive endpoints are also relevant. Therefore, phytoestrogen-containing feeds should be considered not only as nutrient sources, but also as bioactive dietary components whose effects depend on dose, botanical origin and animal physiological status.

### 4.2. Effects on Growth Performance and Carcass Traits

**Figure 6:**
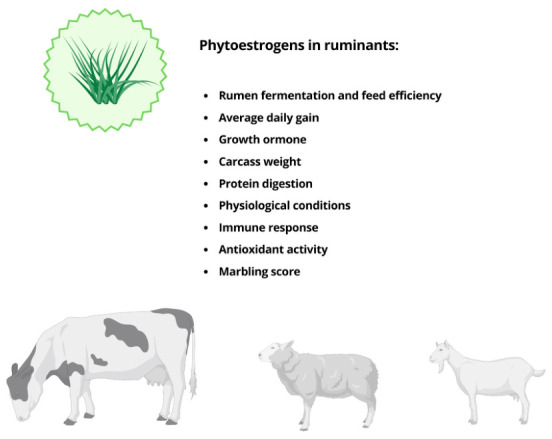
Main effects of phytoestrogens on nutrient utilization and growth performance in ruminants.

The impact of phytoestrogen-rich feeds on growth performance is variable and appears to depend strongly on dose and context. Traditional reports of “clover disease” in sheep and negative effects on fertility did not consistently describe reductions in liveweight gain under grazing conditions, although severe reproductive failures could indirectly affect productivity at the flock level. More recent controlled studies have assessed soybean isoflavone supplementation in growing ruminants. Oral supplementation of soybean isoflavones at moderate doses (e.g., 100 mg/d) modified growth performance and carcass traits in beef cattle, with some studies reporting improved average daily gain, altered non-carcass components and better vascular health in animals grazing endophyte-infected tall fescue (*Lolium arundinaceum* (Schreb.) Darbysh.; syn. *Festuca arundinacea* Schreb.) [98,99]. Isoflavones from red clover hay have also been shown to mitigate ergot alkaloid-induced vasoconstriction in steers grazing toxic tall fescue and to support the recovery of body weight gain during post-grazing periods, suggesting a role in alleviating fescue toxicosis and indirectly sustaining performance [100]. In small ruminants, dietary daidzein and related isoflavones have been associated with an improved growth rate, feed efficiency and immune function in some trials, particularly under suboptimal health or environmental conditions [34,101]. However, results are not entirely consistent, and high phytoestrogen intakes, especially of coumestrol, can negatively affect growth by reducing feed intake or causing health and fertility issues [18,89]. Flaxseed and other lignan-rich feeds are often used primarily to modify the milk fatty acid profile and increase n-3 fatty acid content, but may also influence growth performance depending on inclusion level and processing. Most studies in dairy and beef cattle report no detrimental effects on growth or feed efficiency at typical inclusion rates (5–15% of diet DM), although very high inclusion levels can reduce intake because of an increased fat content [50,78]. Studies evaluating the impact of phytoestrogens on growth and performance in ruminants are summarized in Table 7. In summary, the impact of phytoestrogens on growth performance and carcass traits can be illustrated as depicted in Figure 6.

### 4.3. Effects on Lactational Performance and Milk Composition

Legume forages and by-products containing phytoestrogens can markedly affect milk yield and composition. Dairy cows fed red clover silage generally show higher milk yield and nutrient intake than cows fed grass silage, which has been attributed primarily to the higher digestible energy and protein content of red clover, but may also reflect specific actions of isoflavones and other polyphenols on rumen fermentation and nitrogen metabolism [25,34]. Isoflavone-enriched diets can increase the transfer of daidzein and equol from feed into milk, sometimes without negative effects on milk yield. In an on-farm study with lactating dairy cows, a diet containing higher isoflavone concentrations increased equol content in milk and modified the rumen microbiota, while maintaining or slightly improving milk production and composition [96]. Similarly, feeding cows fresh or ensiled red clover can result in milk equol concentrations in the range of several hundred µg/L, indicating efficient transfer from feed to milk [32]. Flaxseed supplementation, via whole seed, hulls or flaxseed meal, generally increases milk yield or maintains it while improving milk fat composition (higher n-3 fatty acids, conjugated linoleic acid) and enriching milk in enterolactone, with limited evidence of adverse effects on lactation performance when diets are properly balanced [78,80]. Flavonoid-rich citrus and grape by-products can also enhance milk antioxidant capacity and may reduce somatic cell count, although effects on yield are variable and often modest [46,48,51]. In most dairy studies, the primary nutritional concern is not a reduction in milk yield per se, but the potential for phytoestrogen-rich forages (e.g., severely diseased lucerne or clover stands with high coumestrol) to impair fertility, thereby reducing lifetime milk production. These reproductive aspects are discussed in detail in the dedicated section on fertility. Experimental data on the effects of phytoestrogens on milk yield and composition are presented in Table 8.

### 4.4. Effects on Rumen Fermentation, Oxidative Status and Health

Phytoestrogens and related polyphenols can act as modulators of rumen fermentation. Isoflavones from clovers and soybeans have been reported to alter volatile fatty acid patterns, sometimes reducing the acetate:propionate ratio and decreasing methane production, though results are not entirely consistent across studies [50,96]. Red clover isoflavones can inhibit ruminal urease and modulate ammonia dynamics, as noted above, with potential benefits for nitrogen utilization and environmental emissions [97]. A growing body of evidence indicates that phytoestrogen-rich feeds contribute to improved antioxidant status and resilience to stress in ruminants. Phytogenic additives containing isoflavones, flavonoids and other polyphenols have been associated with reduced oxidative stress markers, enhanced activities of antioxidant enzymes (SOD, GSH-Px, CAT) and improved immune parameters in cattle (Table 9).

For instance, resveratrol (a stilbene phytoestrogen) supplementation in heat-stressed dairy cows increased dry matter intake and milk yield and improved indices of thermal comfort and oxidative status, suggesting a protective role under environmental stress [74,110]. In some cases, phytoestrogens may also mitigate specific toxicoses. Isoflavones from red clover have been shown to counteract tall fescue toxicosis by alleviating ergot alkaloid-induced vasoconstriction and improving peripheral blood flow in cattle and goats, thereby supporting feed intake, thermoregulation and performance [99,100]. Such “functional” effects illustrate that, beyond their endocrine activity, phytoestrogens can be considered as bioactive feed components with both risks and potential benefits depending on context. Taken together, the available evidence suggests that phytoestrogens, at nutritionally realistic doses, are compatible with—and sometimes supportive of—good nutritional performance in ruminants, particularly when provided through high-quality legume forages and well-formulated diets. However, their dual nature as both functional nutrients and endocrine-active compounds necessitates careful consideration of species, physiological stage and overall dietary profile, especially in breeding animals and under grazing systems dominated by estrogenic legumes.

## 5. Effects of Phytoestrogens on Reproductive Function in Ruminants

Phytoestrogens can act as endocrine disruptors in ruminants because of their structural similarity to 17β-estradiol and their ability to bind estrogen receptors (ERα, ERβ) and modulate hormone-dependent pathways. Their impact on reproduction ranges from subtle changes in estrous behaviour and luteal function to severe, often irreversible infertility, depending on dose, length of exposure, developmental stage and species. Sheep grazing estrogenic pastures are clearly the most sensitive, whereas cattle and goats are generally less affected at comparable intakes [18,111,112]. Studies documenting reproductive outcomes associated with different phytoestrogen classes in ruminants are summarized in Table 10.

### 5.1. Mechanisms of Endocrine Disruption

Phytoestrogens and their metabolites exert reproductive effects in ruminants primarily through interaction with the estrogen signalling system. Owing to their structural similarity to 17β-estradiol, compounds such as genistein, daidzein, formononetin, coumestrol and the enterolignans can bind ERα and ERβ and act as selective estrogen receptor modulators, with agonist or antagonist activity depending on tissue, receptor subtype and local estradiol concentration [2,18,122]. Among microbial metabolites, S-equol shows particularly high affinity for ERβ and is often more potent than its precursor daidzein, making it a key determinant of systemic estrogenic exposure in species that efficiently produce equol in the rumen [11]. In cattle, a central mechanism involves modulation of uterine prostaglandin secretion and luteal function. Soybean-derived isoflavones and their metabolites, especially equol and p-ethyl-phenol, shift endometrial prostaglandin production towards increased PGF_2_α relative to PGE_2_ and can accumulate in the corpus luteum, where they suppress LH-stimulated progesterone secretion and reduce luteal sensitivity to luteotropic factors [11,115,123,124]. These changes provide a plausible explanation for reduced luteal support and increased early embryonic loss reported in cows receiving high-phytoestrogen diets [11,114]. Experimental work in non-ruminant models shows that phytoestrogens can also interfere with the hypothalamic–pituitary–gonadal axis by altering GnRH and LH secretion and follicular dynamics [11,20], which is consistent with field observations of irregular cycles and silent heats in exposed cattle and sheep [10,18]. Long-term exposure may produce more persistent effects. In sheep, chronic grazing of estrogenic pastures induces structural changes in the cervix and uterus that persist after animals are removed from the offending forage and can result in permanent infertility [111,112,125]. Such lesions resemble organizational effects of excess estrogen during critical developmental windows and are thought to involve epigenetic changes in reproductive tissues, although the underlying mechanisms remain incompletely defined [18,112]. Overall, the molecules of greatest concern for female reproductive function in ruminants appear to be S-equol, p-ethyl-phenol and coumestrol, which combine relatively high ER affinity with specific actions on uterine prostaglandin secretion and luteal progesterone production [112,115,124].

### 5.2. Female Reproduction in Sheep: Estrogenic Pastures and Clover Disease

Sheep are regarded as the ruminant species most sensitive to dietary phytoestrogens. The classical syndrome known as clover disease was first recognized in Australian flocks in the 1940s and was associated with grazing subterranean clover cultivars containing very high concentrations of isoflavones, particularly formononetin and biochanin A [13,111]. Clinical observations and subsequent pathological investigations described a wide range of reproductive disturbances in affected ewes, including reduced conception and lambing rates, temporary or permanent infertility, dystocia, uterine and vaginal prolapse and structural lesions of the cervix and uterus. Abnormal mammary development and altered lactation were also reported [111,112,125]. Adams [125] showed that many ewes grazing estrogenic pastures develop permanent infertility as a consequence of chronic phytoestrogen exposure. Histological examination of reproductive tissues revealed cervical and uterine changes consistent with long-term estrogenic overstimulation and mild sexual transdifferentiation, even in animals without overt clinical signs of clover disease. More recent work has confirmed that ewes exposed to highly estrogenic red or subterranean clover pastures may exhibit altered cervical mucus composition and reduced sperm transport, contributing to subfertility beyond simple mechanical obstruction or gross anatomical abnormalities [112]. Controlled feeding trials support these field observations: Mustonen and Taponen [126] fed nulliparous ewes red clover silage rich in formononetin, biochanin A, genistein and daidzein for several months and reported reduced fertility and increased premature udder development compared with animals receiving grass silage. The risk of reproductive failure in sheep appears to increase markedly when dietary phytoestrogen intake exceeds moderate levels and exposure is prolonged. Recent data indicate that total phytoestrogen concentrations above approximately 500–750 mg/kg dry matter are associated with a higher likelihood of reduced ovulation rate, lower conception, embryonic loss and clinical signs of estrogenism, particularly when older high-estrogen clover cultivars and coumestrol-rich diseased lucerne make a substantial contribution to the diet. Management therefore focuses on reducing exposure of breeding ewes to highly estrogenic pastures during joining and early pregnancy and on lowering the estrogenic potential of the sward, for example by using low-estrogen clover cultivars, avoiding stressed or diseased legume stands that tend to accumulate coumestrol and isoflavones, and periodically testing forage phytoestrogen content in high-risk situations. These measures have substantially reduced the incidence of overt clover disease in modern production systems, but the underlying sensitivity of ewes to phytoestrogens remains a critical constraint when legume-rich swards are used in breeding flocks.

### 5.3. Female Reproduction in Cattle and Goats

Cattle are generally less sensitive to phytoestrogens than sheep, but adverse effects on ovarian function, estrous expression and fertility have been documented when exposure is high or prolonged [11,18]. In dairy cows fed soy-rich diets, the isoflavones genistein and daidzein and their metabolites equol and p-ethyl-phenol have been detected in plasma, uterine tissues and corpus luteum [11,127]. These compounds alter endometrial prostaglandin secretion, increasing PGF_2_α relative to PGE_2_, and can suppress LH-stimulated progesterone secretion by luteal cells, thereby weakening luteal support of early pregnancy [11,123]. Field and experimental studies report associations between high phytoestrogen intake and prolonged or irregular cycles, silent estrus, reduced conception rates and increased early pregnancy loss, especially when high soy concentrates are combined with estrogenic pastures or conserved forages [18,114]. Sensitive categories include high-yielding dairy cows around breeding, embryo donor cows and heifers in early reproductive life [11,18]. Pasture legumes can also compromise reproductive performance in beef cows under specific conditions. In Angus cows grazing mixed-legume pastures with elevated coumestrol and isoflavone levels, reduced superovulatory response and poorer embryo quality have been observed, with embryo quality negatively related to plasma equol and coumestrol concentrations, findings that are particularly relevant for multiple ovulation and embryo transfer programmes [12]. In goats, evidence is more limited, but high intakes of estrogenic lucerne or clover have been associated in some field reports with irregular estrus and lower conception rates, especially when does graze stressed or diseased legume stands [6,18,128]. Overall, although cattle and goats are more tolerant than sheep, the combination of legume-rich forages and soy-based concentrates can create conditions under which phytoestrogens adversely affect female reproductive performance, and exposure of breeding animals to highly estrogenic diets during critical reproductive windows should be avoided [11,18].

### 5.4. Male Reproductive Function

Available evidence indicates that male ruminants are less clearly affected by dietary phytoestrogens than females, although this view is being reconsidered. A recent comprehensive review concluded that in vivo studies have reported few overt fertility failures in bulls at practical levels of exposure, and in some cases phytoestrogen-rich diets or phytogenic additives have been associated with increased scrotal size and higher sperm counts in prepubertal bulls [18]. However, the same authors emphasize that the evidence base remains limited and that subtle or long-term effects cannot be excluded. More recent longitudinal work in rams has demonstrated that low-to-moderate dietary phytoestrogen exposure can transiently disrupt spermatogenesis. Pool and Kent [129] showed that rams grazing estrogenic pastures had specific stages of spermatogenesis impaired, with modest but significant reductions in sperm quality and alterations in seminal plasma proteome; these changes largely resolved after animals were returned to a non-estrogenic pasture. This study provides direct evidence that even physiologically realistic phytoestrogen intakes can compromise male fertility, albeit in a reversible manner.

Experimental studies using bull semen further suggest that phytoestrogens can modulate sperm function at the cellular level. Treatment of frozen–thawed bull sperm with genistein reduced pronuclear formation after in vitro fertilization and decreased sperm motility, even though it also lowered DNA fragmentation, indicating a complex balance between potential protective and detrimental effects on sperm quality [119]. Studies on phytogenic feed additives in male ruminants underline that such compounds may improve some semen and hormonal traits, but also stress the need for more targeted work to clarify safety margins and to avoid high phytoestrogen loads during sensitive periods such as puberty or intensive breeding use [128]. Overall, current data suggest that typical dietary exposures in bulls are unlikely to cause dramatic fertility losses, but the findings in rams and in vitro sperm models justify a cautious approach to feeding highly estrogenic forages or concentrates to breeding males.

### 5.5. Developmental Windows and Transgenerational Aspects

The timing of exposure appears to be as important as the absolute level of phytoestrogens. In sheep, chronic intake of estrogenic clovers during the mating season and early pregnancy has been shown to induce long-lasting structural changes in the cervix and uterus, with many ewes remaining permanently infertile even after they are removed from the offending pastures [112,125]. These lesions resemble organizational effects of excess estrogen during critical developmental windows and suggest that prolonged exposure in adult life can, in practice, mimic developmental programming of the reproductive tract. In cattle, recent work indicates that exposure can begin before birth. Dewulf and Van Eetvelde [130] demonstrated that dams fed isoflavone-rich diets transfer daidzein, genistein and equol to their offspring in utero, with these compounds measurable in calf plasma and hair at birth. Observational studies suggest possible associations between prenatal phytoestrogen exposure, altered anogenital distance and oxidative status, and later reproductive performance in heifers, although causal relationships still need to be confirmed [18,131]. Taken together, these findings indicate that peri-conception, gestation and early postnatal life represent windows of heightened sensitivity in which even moderate phytoestrogen intakes may have disproportionate long-term consequences for reproductive traits, particularly in breeding ewes and replacement heifers.

## 6. Practical Thresholds and Risk Management

Clear-cut safe thresholds for phytoestrogen intake in ruminants are difficult to define, because risk depends not only on dietary concentration but also on duration and timing of exposure, plant species and the physiological status of the animal. Nevertheless, current evidence supports some practical ranges and management rules of thumb. Reviews of grazing livestock indicate that sheep show reproductive disturbances at comparatively low phytoestrogen intakes, especially when grazing older subterranean clover cultivars or stressed lucerne and clover stands that accumulate high levels of formononetin, biochanin A and coumestrol [18,111]. Long-term exposure under these conditions can lead to permanent infertility in ewes, even when estimated total phytoestrogen concentrations are only in the mid-range compared with extreme clover-disease situations. Other authors suggest that, in both sheep and cattle, total dietary phytoestrogen concentrations above roughly 500–750 mg/kg dry matter are increasingly associated with a reduced ovulation rate, lower conception and increased embryonic loss, particularly when the contribution from estrogenic legumes is high and exposure coincides with mating or early gestation [11]. However, these values should be regarded as indicative rather than absolute cut-offs, and more conservative limits are advisable for breeding ewes, embryo donors and high-producing dairy cows. From a practical standpoint, risk mitigation focuses on reducing the estrogenic potential of the forage base and avoiding high exposure during critical reproductive windows. Key strategies include the use of low-estrogen cultivars of clover and lucerne, limiting the time breeding animals spend on highly estrogenic pastures around joining and early pregnancy, and moderating soybean inclusion in diets where legume forages already contribute substantial phytoestrogen loads [11,111]. Recent data also show that haymaking and ensiling practices can be used to lower phytoestrogen content: extended wilting of red clover hay and silage reduced phytoestrogen concentrations while preserving nutritional value, providing a useful tool to decrease dietary exposure without abandoning legumes altogether [132]. Overall, a careful choice of cultivars, targeted forage conservation and strategic grazing management allow producers to harness the nutritional benefits of legumes and other phytoestrogen-containing feeds while minimizing their negative impact on reproductive performance.

## 7. Conclusions

Phytoestrogens are ubiquitous in ruminant diets based on legume forages, soybean products and flaxseed, and should be considered bioactive feed components. At realistic inclusion levels, isoflavones, lignans, coumestans and selected flavonoids can exert beneficial effects by supporting intake, rumen fermentation, nitrogen utilization, antioxidant status and immune function, and by contributing to milk yield and product quality. The same compounds, however, can act as endocrine disruptors. Sheep are clearly the most sensitive species, and prolonged exposure to highly estrogenic clovers or diseased lucerne can lead to marked reductions in fertility and, in extreme cases, permanent infertility. In cattle and goats, the safety margin appears wider, but high phytoestrogen intake from combinations of legume-rich forages and soy-based concentrates has been associated with altered prostaglandin secretion, impaired luteal function and increased risk of early embryonic loss in susceptible categories such as high-yielding dairy cows, heifers and embryo donors. Risk is not defined by a single threshold but by phytoestrogen class and metabolite profile, plant species and cultivar, agronomic and conservation practices, dose and duration of exposure, and species and physiological state. Practical control relies on cultivar choice, pasture and disease management, conservation practices and moderation of soy inclusion, particularly around mating and early gestation. The available evidence also highlights gaps for goats, buffalo, breeding males and developmental exposure; future research should address these areas and refine intake guidelines that allow the use of phytoestrogen-rich feeds while safeguarding reproductive efficiency and animal health.

## Figures and Tables

**Table 1:** Classification of phytoestrogens [1,11,18,19,20].

Isoflavones	Flavonoids	Stilbenes	Coumestans	Lignans
Genistein	Flavones	Resveratrol	Coumestrol	Secoisolariciresinol
Daidzein	Flavanones		4′-methoxy-coumestrol	Matairesinol
Formononetin	Chalcones		3′-methoxy-coumestrol Trifoliol	Pinoresinol
Biochanin A			Sativol	Lariciresinol
			Medicagol	
			Lucernol	
			Repensol	
			11,12-dimethoxy-7-hydroxy	

**Table 2:** The isoflavones and their corresponding glycosides molecular architecture [11,20].

Aglycon	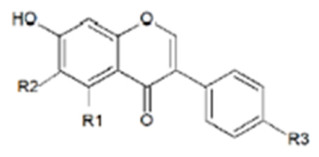	R1	R2	R3	
Daidzein		H	H	OH	
Glycitein		H	OCH_3_	OH	
Genistein		OH	H	OH	
Formononetin		H	H	OCH_3_	
Biochanin A		OH	H	OCH_3_	
Glucoside			R4	R5	R6
Daidzin	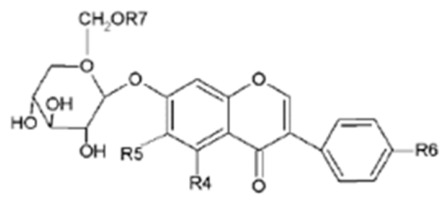	H	OH	H	R7
Genistin	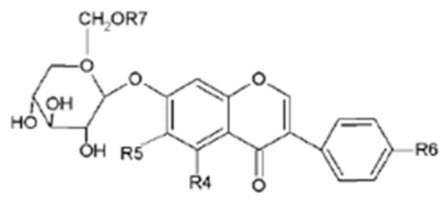	H	H	OH	H
Ononin		H	H	OCH_3_	H
Glycitin		H	OCH_3_	OH	H
Acetyldaidzin		H	H	OH	COCH_3_
Sissotrin		OH	H	OCH_3_	H
Acetylglycitin		H	OCH_3_	OH	COCH_3_
Acetylgenistin		OH	H	OH	COCH_3_
Malonylgenistin		OH	H	OH	COCH_2_COOH
Malonyldaidzin		H	H	OH	COCH_2_COOH
Malonylononin		H	H	OCH_3_	COCH_2_COOH
Malonylglycitin		H	OCH_3_	OH	COCH_2_COOH
Malonylsissotrin		OH	H	OCH_3_	COCH_2_COOH

**Table 3:** Profile and concentration of isoflavones in specific plant species [1].

Plant Species	Main Isoflavones	Isoflavone Content
Alfalfa (*Medicago sativa*)	Coumestrol, formononetin	0.5–3.5%
Red clover (*Trifolium pratense*)	Genistein, daidzein, formononetin, biochanin A	1.5–2.5%
Mung bean (*Vigna radiata*)	-	3.51 mg/kg crude sample
Psoralea (*Psoralea corylifolia*)	-	2 g/kg dried sample
Kudzu root (*Pueraria lobata*)	Puerarin, daidzein, genistein	0.95 g/kg daidzein
Soybean (*Glycine max*)	Daidzein, genistein, glycitein	0.1–0.5%
Chickpea (*Cicer arietinum*)	Daidzein, genistein, glycitein	-

- indicates that the compound or concentration was not reported/not available in the original source.

**Table 4:** Isoflavones and lignans concentrations in different food sources are expressed in nanomoles per gram of dry weight. These values, sourced from Cassidy [27], were obtained through the method of isotope dilution gas chromatography–mass spectrometry utilizing selected ion monitoring, as detailed in Dixon [28].

Plant Species	Daidzein	Genistein	Secoisolariciresinol	Matairesinol
Soybean	413–2205	993–3115	<1–8	<1
Sunflower seed	<1	<1	17	0
Kidney bean	<1–2	<1–19	2–4	<1
Wheat bran	<1	<1	3	0
American groundnut	<1	4–30	<1–2	<1
Rye bran	0	0	4	5
Chickpea	<1–8	3–8	<1	0
Cranberry	0	0	29	0
Pea	<1	<1	<1	<1
Raspberry	0	0	4	0
Lentil	<1	<1	<1	<1
Broccoli	<1	<1	11	<1
Kudzu root	7283	467	<1	<1
Zucchini	0	0	23	<1
Flaxseed	0	0	10,247	30
Beetroot	0	0	3	<1
Sesame seed	6	<1	2	17
Black tea	Trace	Trace	73	12
Peanut	1	2	8	<1
Barley (whole grain)	<1	<1	2	0
Strawberry	0	0	33	<1
Blueberry	0	0	23	0
Red cabbage	<1	<1	4	<1
Garlic	0	0	11	<1
Carrot	0	0	10	<1
Green tea	Trace	Trace	75	5

Trace = detected only in trace amounts but not quantified in the original source; <1 = concentration below 1 nmol/g dry weight.

**Table 5:** Various structural forms are observed within specific plant families [57].

Stilbene	Occurrence	R3	R5	R3′	R4′	
Trans-piceid	Vitis	OGlu	OH	H	OH	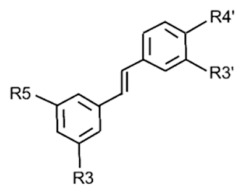
Trans-resveratrol	Vitis, Arachis, Fallopia	OH	OH	H	OH	
Piceatannol	Picea	OH	OH	OH	OH	
Trans-pterostilbene	Vitis, Vaccinium	OCH_3_	OCH_3_	H	OH	
Pinosylvin	Pinus	OH	OH	H		
Pinosylvin monomethyl ether	Pinus, Alnus	OCH_3_	OH	H	OH	
Rhapontin	Rheum	OGlu	OH	OH	OCH_3_	
Astringin	Picea	OGlu	OH	OH	OH	

**Table 6:** Lignan content and composition in certain plant species [1].

Plant Species	Main Lignans	Lignan Content Per 100 g
Flax seed (*linum usitatissimum* L.)	Secoisolariciresinol, matairesionol	0.3 g
Sesame seed (*sesamum indicum* L.)	Sesamolin	29 mg
Cabbages	-	185–2321 µg
Cereal’s grain (rye, barley, wheat)	Syringaresinol	-
Fruit (berries and nuts)	Medioresinol	-

- indicates that quantitative values were not available or not reported in the original source.

**Table 7:** Effects of estrogenic compounds on production.

Estrogenic Compounds	Species	Main Finding	Reference
Genistein	Ewes and lambs	Increased daily weight gain	[102]
Daidzein	Beef cattle	Enhanced rumen fermentation in beef cattle	[103]
Daidzein	Steers cattle	Improve the marbling score and enhance the intramuscular fat content	[104]
Equol	Lactating cows	Microbial richness decreased	[96]
Isoflavones	Cows	Increased feed intake	[105]
Biochanin A	Dairy cows	Enhanced nitrogen utilization efficiency	[106]
Isoflavones	Lambs	Internal fat and total fat percentages	[102]
Daidzein	Bull calves	Promoted the digestion of dietary proteins	[107]
Daidzein	Steers	Increased marbling score and the intramuscular fat content	[104]
Formononetin	Lambs	Increased body weight gain	[102]

**Table 8:** The effects of estrogenic compounds on milk yield and composition.

Estrogenic Compounds	Species	Main Finding	Reference
Equol	Dairy cows	Increased milk production	[108]
Biochanin A	Dairy cows	Increase milk production	[106]
Biochanin A	Dairy cows	Decreased nitrogen level in milk	[106]

**Table 9:** The effects of estrogenic compounds on the immune system.

Estrogenic Compounds	Species	Main Finding	Reference
Biochanin A	Dairy cows	Did not adversely affect body condition or general health	[106]
Daidzein	Bull calves	Increases serum levels of IgG, IgA and IgM	[107]
Genistein	Bovine	Inhibits bovine viral diarrhea virus	[109]

**Table 10:** The effects of estrogenic compounds on reproduction.

Estrogenic Compounds	Species	Main Finding	Reference
Daidzein	Cow	Infertility	[11]
Isoflavones	Goat	Increased testosterone concentrations	[113]
Genistein	Bull	Decreased fertility of bulls	[114]
Equol	Heifer	Embryonic loss happened	[115]
Coumestrol	Heifer	Vaginal prolapse has been observed	[116]
Coumestrol	Cattle	Increased number of immature oocytes	[117]
Genistein	Cattle	Increased number of immature oocytes	[117]
Coumestans	Ewe	Decreased the ovulation rate	[118]
Formononetin	Ewe	Prolapse of the cervix, vagina and the rectum	[102]
Genistein	Bull	Reduced sperm motility	[119]
Genistein	Male	Decreased acrosome reaction	[120]
Genistein	Ram	Decreased sperm motility	[121]

## Data Availability

Not applicable.
